# Anodisation Increases Integration of Unloaded Titanium Implants in Sheep Mandible

**DOI:** 10.1155/2015/857969

**Published:** 2015-09-08

**Authors:** Warwick J. Duncan, Min-Ho Lee, Tae-Sung Bae, Sook-Jeong Lee, Jennifer Gay, Carolina Loch

**Affiliations:** ^1^Sir John Walsh Research Institute, Faculty of Dentistry, University of Otago, Dunedin, New Zealand; ^2^Department of Dental Biomaterials and Institute of Biodegradable Materials, Institute of Oral Bioscience and BK21 Plus Project, School of Dentistry, Chonbuk National University, Jeonju, Republic of Korea; ^3^Department of New Drug Discovery and Development, Chungnam National University, Daejeon, Republic of Korea

## Abstract

Spark discharge anodic oxidation forms porous TiO_2_ films on titanium implant surfaces. This increases surface roughness and concentration of calcium and phosphate ions and may enhance early osseointegration. To test this, forty 3.75 mm × 13 mm titanium implants (Megagen, Korea) were placed into healed mandibular postextraction ridges of 10 sheep. There were 10 implants per group: RBM surface (control), RBM + anodised, RBM + anodised + fluoride, and titanium alloy + anodised surface. Resonant frequency analysis (RFA) was measured in implant stability quotient (ISQ) at surgery and at sacrifice after 1-month unloaded healing. Mean bone-implant contact (% BIC) was measured in undemineralised ground sections for the best three consecutive threads. One of 40 implants showed evidence of failure. RFA differed between groups at surgery but not after 1 month. RFA values increased nonsignificantly for all implants after 1 month, except for controls. There was a marked difference in BIC after 1-month healing, with higher values for alloy implants, followed by anodised + fluoride and anodised implants. Anodisation increased early osseointegration of rough-surfaced implants by 50–80%. RFA testing lacked sufficient resolution to detect this improvement. Whether this gain in early bone-implant contact is clinically significant is the subject of future experiments.

## 1. Introduction

Implant dentistry has become a common option for oral rehabilitation treatments for partially and fully edentulous patients. However, the clinical success of oral implants is still directly related to their early osseointegration. The establishment of direct bone-implant contact without an intervening connective tissue layer is a fundamental prerequisite for implant-supported prostheses and their long-term success [1].

Due to its excellent mechanical properties, biocompatibility, and corrosion resistance, titanium and titanium alloys are widely used in orthopaedic and dental implants. Most dental implants are made from grade 4 c.p. Ti, while their alloys are mainly composed of Ti-6Al-4V, which possess greater yield strength and fatigue properties than pure titanium [1, 2]. In the mid-1980s, Ti-6Al-7Nb alloys were also introduced into clinical use as a substitute for Ti-6Al-4V, due to higher biocompatibility and lower cost of niobium compared to vanadium [3]. One of the key features of titanium and titanium alloy implants is their oxide passive layer which is typically 2 to 5 nm thick. This layer is responsible for the well-documented corrosion-resistance property of titanium [2].

Bone osseointegration is directly dependent on both biomechanical interlocking and biological interactions through biochemical bonding. Biomechanical interlocking is thus favoured by the surface irregularity and roughness of dental implants. Different surface modification techniques have been developed to alter the surface topography of implants and increase their short- and long-term success. These include mechanical methods (e.g., sandblasting), chemical methods (e.g., acid etching), and special coatings (e.g., plasma spraying) [2]. Currently, modifications on the surface roughness of titanium implants have been sought to produce nanosized surface features in order to improve osseointegration and biomechanical fixation, mainly because bone is also a nanostructured material. Recent studies showed that an artificial increase in the thickness of the native oxide layer results in stronger and more effective bone response [4–8].

Anodisation or anodic oxidation is a well-established electrochemical method to promote surface modification in valve metals, increasing the thickness of protective layers to more than 1000 nm [2]. The dissolution of the oxide layer along the current convection lines generates micro or nanopores on the titanium surface. Anodised surfaces promote a strong reinforcement of the bone response with higher values for biomechanical and histomorphometric tests in comparison to machined surfaces [1, 4–8]. Anodised implants also show a higher clinical success rate [9].

Together with the mechanical interlocking through bone growth in pores, biochemical bonding is also a fundamental outcome of surface treatment of implants. Chemical treatments involving fluoride solutions have been proven to create surface roughness and fluoride incorporation favourable to the osseointegration of dental implants by rendering a bioactive implant surface [1, 10]. An increase in surface concentrations of calcium and phosphate ions is also proven to increase biocompatibility and reinforce bone tissue reactions of electrochemically oxidized titanium implants [8]. In anodised surfaces prepared using electrolytes containing Ca and P, such as calcium glycerophosphate (Ca-GP) and calcium acetate (CA), both Ca and P contained in the oxide layer achieve a Ca/P ratio close to hydroxyapatite (1.67), which may be an important factor for biomechanical bonding with bone tissues [2].

Anodisation is considered a quick and efficient modification method for titanium implants which shows significant promise for enhancing their lifetime [2]. Nevertheless, the exact role of surface chemistry and topography on the early events of the osseointegration of dental implants remains poorly understood.* In vitro* and* in vivo* studies have been pursued to elucidate the efficiency of anodisation treatments in comparison to machined surfaces [11–13]. Many animal models have been employed for intra- and extraoral healing of bone around metal implants, including well-established models of bone healing using sheep [14]. Among the advantages of this model in biomedical research are the similarities in size, weight, and general physiology between sheep and humans, as well as their easy handling and robust recovery from anaesthesia and surgery. Similarities in bone structure and mineralisation rates between humans and sheep are also an advantage, together with good knowledge of healing rates in sheep bone [14].

This study aims to elucidate the* in vivo* effects of anodisation on commercially available sandblasted (RBM) implants treated with hydrothermal anodic oxidisation. In particular, we investigated whether anodisation affected bone integration around titanium implants using biomechanical and histomorphometric analyses. An established animal edentulous mandible model using domestic sheep was employed to address these questions.

## 2. Material and Methods

### 2.1. Implants

**Figure 1:**
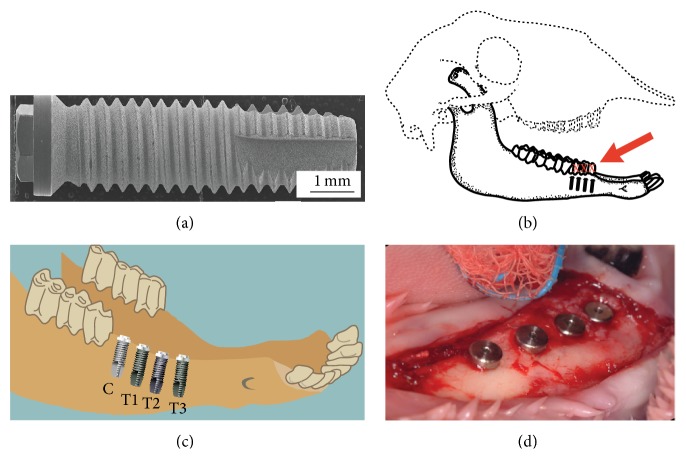
(a) SEM image of a control (resorbable blasting media) implant. (b) Schematic view of a sheep skull showing the site for teeth extraction and future placement of implants. (c) Location of control and test implants placed in the mandible. (d) Implants placed into an edentulous sheep mandible after surgery.

Forty threaded titanium dental implants measuring 3.75 mm diameter × 13 mm long (Megagen Ltd., Korea) were used in this study (Figure 1(a)). Implants had roughened surfaces prepared using resorbable blasting media (RBM) surfaces. Ten of these implants were used as controls, while 30 were treated using anodic oxidisation following Park et al. [15]. An electrolyte solution was prepared by dissolving 0.02 mL/L of DL-*α*-glycerophosphate disodium salt hydrate (DL-*α*-GP) and 0.2 mL/L of calcium acetate (CA) in distilled water. The anodic oxidation was operated using a direct current (DC) regulated power supply employing constant current mode up to the configured voltage. The implants and platinum plate were connected to both anode and cathode sides of the DC regulated power source and were treated with 30 mA/cm^2^ and 300 V. Voltage was kept constant during the electrical treatment. The subsequent hydrothermal treatment was performed in an autoclave using distilled water at 300°C and 8.8 MPa of steam pressure. There were four experimental groups in this study, all of them consisting of ten implants (Table 1).

### 2.2. Scanning Electron Microscopy and EDX Analysis

Scanning electron micrographs were obtained in a JSM-5900 SEM (JSM-5900, JEOL, Tokyo, Japan) operating at 20 KV. Magnifications ranged from 100 to 3000x. Samples were sputter-coated with platinum (ion sputter, E-1020, Hitachi) for observation of the surface morphology. The chemical composition of the surface coating was analyzed with an energy dispersive spectroscope (EDX, Oxford) incorporated into the scanning electron microscope with an acquisition time of 2 min.

### 2.3. Dental Extraction Surgery

Ethical approval for the surgical procedures in this study was obtained from the University of Otago Animal Ethics Committee (approval number AEC74-06). Ten Romney cross ewes aged four years were purchased from the same flock. Prior to surgery, animals were starved overnight and had antibiotics administered (penicillin/streptomycin 3 mL/kg I.M) and general anaesthesia was induced with thiopentone 20 mg/kg i.v. The sheep were intubated orally and anaesthesia was maintained by halothane (1-2%) and nitrous oxide/oxygen in a ratio of 1 : 2. Extraction of premolar teeth followed an atraumatic approach. A flap was raised around the mandibular premolars and teeth were loosened with periotomes and dental elevators. Premolars were then sectioned with a tungsten carbide tapered fissure bur and fully removed in pieces. Incisions were closed with resorbable sutures (Dexon 3/0, Ethicon Inc., New Jersey). An antiseptic mouthwash was applied after surgery (chlorhexidine, 10 cc 0.2% aq.) for three days, and animals were returned to normal grazing. Healing time for dental extractions was set as three months.

### 2.4. Implant Placement Surgery

Four implants (one control and one from each test group) were placed into healed mandibular postextraction ridges of each of the ten sheep (Figures 1(b) to 1(d)) under general anaesthetic. A standard surgical procedure for implant placement was adopted under sterile conditions at all times. A flap was raised in the region where the mandibular premolar teeth were extracted. Osteotomies were then created in the healed postextraction ridges in the mandibles using the manufacturer's drills and the implants were placed at this site at low speed with copious chilled saline irrigation. Cover screws were fitted in the implants and the incisions were closed with resorbable sutures. 2 mL of a long-acting local anaesthetic (bupivacaine hydrochloride 5.0 mg/mL, Astra Zeneca, New Zealand) was injected into the surgical site to minimize postoperative discomfort. The sheep were transferred to a postsurgery recovery area where they were closely monitored by veterinary staff for three days. Postoperative anti-inflammatory (carprofen 4 mg/kg s.c., Pfizer Animal Health, New Zealand) and antibiotic agents (streptopen 5 mL, Glaxo Animal Health, New Zealand) were applied as required. The sheep had their mouth syringed with antiseptic mouthwash after surgery. The animals were then returned to normal grazing on pasture for the designated healing period.

One month after unloaded healing, all animals were euthanised. Under general anaesthetic, animals had the carotid artery and jugular veins exposed bilaterally. The carotid artery was then canulated and the animal was perfused with heparinised saline solution followed by 10% formalin. The mandibular site was dissected* en bloc* prior to biomechanical testing.

### 2.5. Biomechanical Testing

Resonance frequency analysis (RFA) was performed for each implant using the Mentor II device (Osstell Mentor, Integration Diagnostics Inc., Sweden). RFA values were measured in implant stability quotient (ISQ) and were obtained in pairs at right angles to each other (buccolingually and mesiodistally). Mean RFA values were obtained for each implant both at surgery and after sacrifice of the animals.

### 2.6. Histomorphometric Analysis

All specimens were further fixed in formalin and dehydrated in ascending concentrations of ethanol. Specimens were then cleared in xylol and embedded in methylmethacrylate. Embedded blocks were sectioned on a Struers Accutom-50 precision cut-off saw (Struers, Copenhagen, Denmark) using a R330 diamond wafered wheel under water irrigation. Sectioned slices were glued to plastic slides using cyanoacrylate glue and polished on the Struers TegraPol-21/TegraForce 5 system (Struers, Copenhagen, Denmark) using silicon carbide paper (1200 to 4000 grit). At the end of the process, specimens had an average thickness of 80–100 *μ*m.

Mounted slides were surface stained with one part of MacNeal's tetrachrome (methylene blue, azur II, and methyl violet) followed by two parts of toluidine blue. Stained sections were viewed using an Olympus Vanox-T microscope (Olympus Australia Pty Ltd., Australia) at 20x magnification and digital images were captured using a Diagnostic Instruments SPOT RT Colour camera (SciTech Pty Ltd., Australia). Normally the two most central sections from each implant were chosen for analysis, and buccal and lingual surfaces were measured separately. After the best three consecutive threads from each side of the implant were identified [16, 17], the bone-to-implant contact (BIC) within each thread was measured in calibrated images using ImageJ software (National Institutes of Health, Bethesda, USA). BIC was expressed as a percentage of the total perimeter of each thread. BIC results for each implant comprised mean values and standard deviation obtained in the three best threads for both tests (Tests 1, 2, and 3) and control (C) groups.

## 3. Results

### 3.1. Scanning Electron Microscopy and EDX Analysis

**Figure 2:**
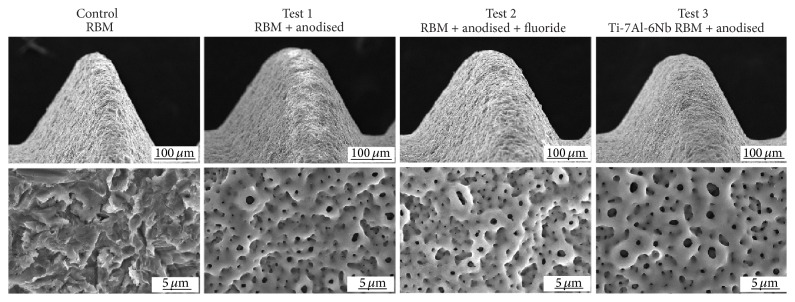
SEM images of control (resorbable blasting media) and test (anodic oxidised RBM) surfaces. Low power views: magnification 200x. High power views: magnification 3000x.

Scanning electron micrographs of the surfaces demonstrated the roughening caused by the resorbable blasting media and showed the modulation of this surface following anodic deposition and hydrothermal treatment. Such modulation was not observed in the control implants (Figure 2).

Energy dispersive X-ray analysis of control and test implant surfaces evidenced the incorporation of O, P, and Ca into anodised surfaces (Table 2). Minor traces of V were observed in Tests 2 and 3 implants. Al and Nb were also present in Test 3 implant (alloy).

### 3.2. Clinical Results

Only one implant among the 40 used showed evidence of failure, providing a survival rate after one month of 97.5%. The failed implant was one of the controls placed in the most anterior position of the lower jaw, which presented less than 5% BIC.

### 3.3. Resonant Frequency Analysis

**Figure 3:**
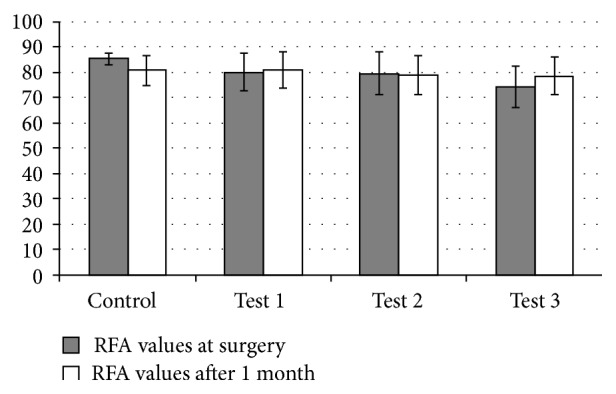
Mean RFA values (±SD) at surgery and after one-month healing for control and test implants.

Mean resonant frequency values differed between groups at surgery (Friedman's *χ* ^2^ = 0.02), with Test 3 implant showing statistically significant lower mean values than control implants. Implant stability quotient (ISQ) for control implants averaged 85.5, while ISQ values for Test 3 implants averaged 74.4 (*p* = 0.005). There was no statistically significant difference in RFA mean values measured after 1-month healing (Friedman's *χ* ^2^ = 0.4). There was a nonsignificant trend for increasing RFA values after 1-month surgery for Test 1 and Test 3 implants, but not for controls and Test 2 (Figure 3).

### 3.4. Descriptive Histology

All implants showed signs of osteoconduction and integration after one-month healing with no evidence of inflammation or titanium particles found within the tissue. Control specimens had large masses of disorganized calcified material filling the thread spaces which appeared to be resorbed bone tissue or residual debris. Test 3 implants had consistent evidence of resorption of this debris with osteoconduction of new bone that followed and largely filled the threads. Tests 1 and 2 implants showed a similar picture, with less debris than the control implants, largely incorporated into new bone, but with less new bone filling the threads.

### 3.5. Histomorphometric Analysis

**Figure 4:**
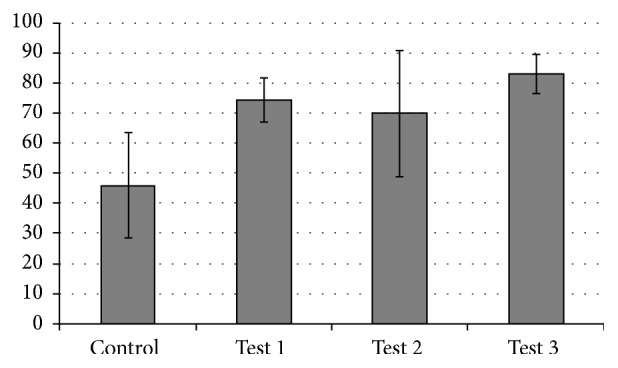
Mean values of bone-implant contact (% BIC) (±SD) in the best three consecutive threads of control and test implants.

**Figure 5:**
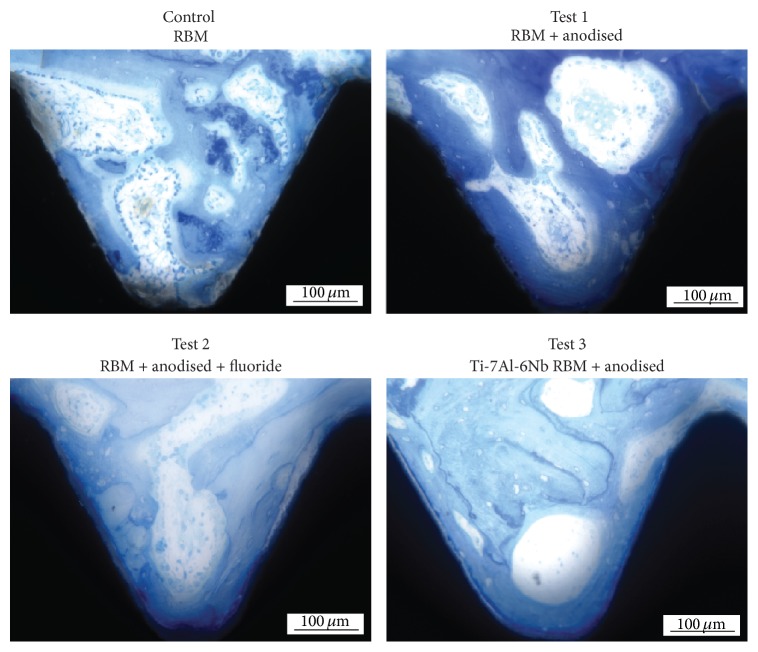
Histological images of bone integration showing the bone tissue in the threads on control and test implants (magnification 20x).

Average values of bone-implant contact (% BIC) in the best three consecutive threads showed that there was a marked difference in % BIC between control and test implants after 1-month healing (Figure 4). All test implants had statistically significant higher % BIC compared with the control implant (Friedman's *χ* ^2^ = 0.0). Anodisation nearly doubled bone-implant contact for all test groups, with an increase in bone-implant contact ranging from 50% (Test 2) to 80% (Test 3 implants) (Figure 5). Individual comparisons using Wilcoxon signed ranks test showed statically significant differences in % BIC for all pairs analysed, with exception of Test 1 (RBM + anodised) and Test 2 (RBM + anodised + F-) implants which were statistically similar (*p* = 0.7).

## 4. Discussion

This study aimed to investigate the effects of anodisation of titanium implants on bone integration using biomechanical and histomorphometric analyses. While resonant frequency analysis did not show evidence of changes in implant stability quotient between control and test implants, histomorphometric analysis revealed significant increase in bone-implant contact of anodised in comparison to sandblasted implants.

The overall clinical success of this study was of 97.5%, with only one implant showing signs of failure. This implant success rate is similar to other studies which compared anodised and machined/sandblasted implants in animal models and also in clinical studies with humans [18, 19]. Jungner et al. [9] reported an overall clinical success of 98.2% in a study with 136 patients, with all cases of failed implants recorded for turned surfaces in contrast with no failure for anodised implants. In the present study, the only failed implant was also a control implant, which suggests higher clinical success and stronger bone response for anodised implants.

Chemical analysis of implant surfaces using EDX revealed the incorporation of O, P, and Ca into anodised surfaces, with minor traces of V observed in Tests 2 and 3 implants. In addition, as expected, Test 3 implants also had Al and Nb in its composition. Other studies have reported the chemical composition of the implants studied using different analytical techniques, such as X-ray photoelectron spectroscopy (XPS) [5, 6, 8, 20], which makes the comparison with the findings of the present study difficult. EDX and XPS are equally suitable for accurate determination of the mean atomic composition of surface areas. However, XPS analysis can determine the chemical state of detected elements after chemical modification, being more commonly used in chemical analysis of implant surfaces [21].

Biomechanical testing using resonant frequency analysis (RFA) showed that Test 3 implants had lower implant stability quotient values (ISQ) than control implants at surgery, but no statistically significant differences were detected among implants after 1-month healing. When comparing mean average ISQ values for each implant type, results obtained at surgery and after 1-month healing were considerably similar and no clear trend could be detected. These results suggest that resonant frequency analysis lacked sufficient resolution to detect biomechanical changes due to osseointegration in the present study. While some studies using RFA have showed statistically significant increase in implant stability from placement to 4–6-week healing [12, 13], others have shown no significant differences [4]. However, it is known that ISQ values often do not correlate with histomorphometric analyses and bone osseointegration [22]. In a study using an animal model with loaded implants, Al-Nawas et al. [23] found that ISQ values at the start of the loading period were not predictive of implant loss during loading, suggesting that caution should be adopted when using RFA analysis to evaluate the success of implant systems.

Qualitative histological observation showed evidence of osteoconduction in all implant surfaces analysed; however, control implants had large masses of disorganised calcified material between the threads. Test implants, on the other hand, showed less evidence of this resorbed debris and more osteoconduction of new bone tissue. Similar histological analyses conducted in control and modified implants by other researchers also corroborate spread apposition of more homogenous and densely mineralised bone both in contact with the oxidised surfaces and also inside the threaded area of modified implants [6, 8, 24, 25]. Ivanoff et al. [24] reported a higher incidence of inflammatory cells and nonresorbed remnants of old bone entrapped in the bone tissue in contact with control surfaces in contrast to oxidised surfaces. The results of qualitative histological observations obtained in this study and in previous investigations suggest that anodised surfaces are more osteoconductive than nonmodified surfaces.

Histomorphometric analyses showed a marked increase in bone-implant contact of anodised (test) surfaces in comparison to control implants. The early osseointegration of rough-surfaced implants was increased by 50–80% when implants were treated with anodic oxidation. Mean values of % BIC ranged from 46% in control implants to 83% in Test 3 implants. Other studies using anodised surfaces have reported % BIC values ranging from 43% after 1.5-month healing [6], 57% after 3-month healing [11], 58% after 3-month healing [26], 60–70% after 2-month healing [27], 71% after 2-month healing [28], and 83% after 12-month healing [29]. % BIC results of previous research in sheep mandibular models ranged from 44% for machined surfaces and 66% for blasted surfaces [30], to 64% (Osseotite) [31], 69% (TiUnite) [16], 73% (SLA) [30], and 85% (plasma-sprayed HA) [30], after 3-month healing. Our anodic oxidised test surfaces achieved similar results to the best of these after only one-month healing. However, whether this gain in early bone-implant contact is clinically significant in the context of early occlusal loading is still the subject of subsequent experiments.

It is known that electrochemical anodisation using organic electrolytes containing fluoride produces self-organised nanopore structures with higher diameter and length [10, 32]. This modification of the surface roughness is thought to improve osseointegration [1]. In this study, the addition of fluoride to the electrolyte solution did not significantly increase the % BIC of Test 2 implants (anodised surface + fluoride) in comparison to the other anodised test implants. In fact, Test 2 implants had the lowest % BIC of all three surface-modified test implants, suggesting that the addition of fluoride did not result in improved osseointegration.

The test implant with the best % BIC results was Test 3, which comprised a Ti-6Al-7Nb implant with anodised surface. The use of titanium alloys in surgical implant operations has increased in recent years, due to superior material properties and enhanced biocompatibility when compared to pure titanium implants [33, 34]. Lavos-Valereto et al. [33] reported effective biocompatibility of Ti-6Al-7Nb implants surgically inserted into the jaw of dogs, with good levels of osseointegration and bone anchorage between the implant and bone surface. Lee et al. [34] investigated crestal remodelling and osseointegration of c.p. titanium versus Ti-6Al-7Nb implants also using a canine model and found no significant differences between implant technologies for any of the parameters assessed. It is not clear whether the superior results of Test 3 implants (anodised Ti-6Al-7Nb) in this study are reflecting the chemical composition of the alloy, the effect of anodisation, or a combination of both factors.

In summary, bone tissue early integration was significantly stronger for the anodic oxidised implants compared with controls in an animal edentulous mandible model using domestic sheep one month after implant insertions. In particular, anodised Ti-6Al-7Nb implants showed the highest % BIC values of all tested surfaces, although resonant frequency analysis lacked sufficient resolution to detect this improvement. Future studies will determine whether this gain in early bone-implant contact is clinically significant in the context of early occlusal loading.

## Figures and Tables

**Table 1:** Experimental design and groups adopted in this study.

Control	Test implants		
	Test 1	Test 2	Test 3
CP-titanium implant with RBM surface	CP-titanium implant with RBM + anodised surface	CP-titanium implant + RBM + anodised + fluoride	Ti-6Al-7Nb implant with RBM + anodised surface
*n* = 10	*n* = 10	*n* = 10	*n* = 10

**Table 2:** Results of elemental analysis (element and atomic percentages) of implant surfaces using EDX.

	Element	Element %	Atomic %
Control RBM	Ti K	100	100
Total		100	100

Test 1	O K	17.8	38.54
	P K	2.34	2.61
	Ca K	7.59	6.56
	Ti K	72.28	52.29
Total		100	100

Test 2	O K	11.85	28.03
	P K	2.31	2.83
	Ca K	8.78	8.29
	Ti K	75.92	60
	V K	1.15	0.85
Total		100	100

Test 3	O K	21.73	44.22
	Al K	2.16	2.61
	P K	2.92	3.07
	Ca K	11.07	8.99
	Ti K	58.09	39.49
	V K	0.70	0.45
	Nb L	3.33	1.14
Total		100	100
